# Supplementary material for: A Glycolipidated-liposomal peptide vaccine confers long-term mucosal protection against Streptococcus pyogenes via IL-17, macrophages and neutrophils
Source: Nat Commun. 2023 Sep 25;14:5963. doi: 10.1038/s41467-023-41410-7 (PMC10520070; doi:10.1038/s41467-023-41410-7)
Supplement: Supplementary file 1 — Supplementary Information [file 41467_2023_41410_MOESM1_ESM.pdf]

SUPPLEMENTARY INFORMATION

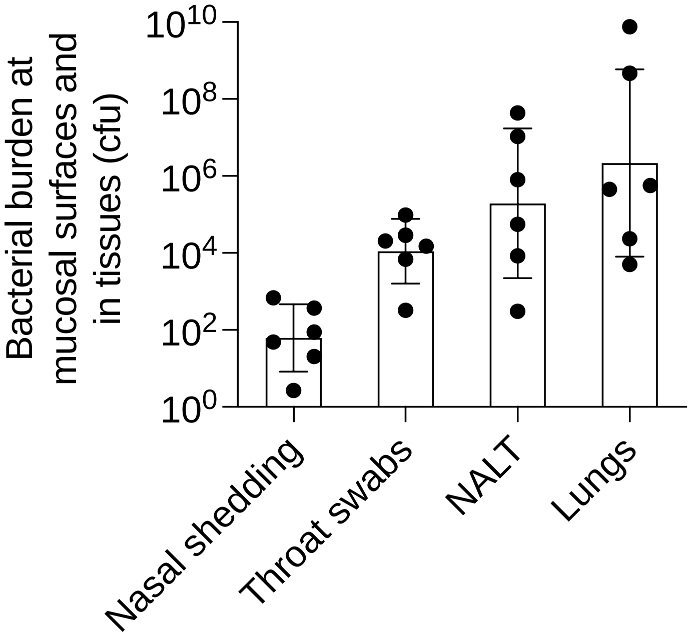

Supplementary Figure 1: Representative data from multiple URT challenge experiments with *S. pyogenes* (*emm* 1) in BALB/c mice. The 6 experiments included in this figure (each dot represents the geomean from one experiment) represent the control groups from other figures in this paper (n=10 mice/group). The mice were infected via the URT with $5x10^6$ cfu/mouse of *S. pyogenes* 2031. Throat swabs (TS) and nasal shedding (NS) were collected on days 1-3 post-challenge. For TS, the throats were swabbed using a flocked swab (Copan Diagnostics, USA). Swabs were then suspended in PBS, serially diluted and dot-plated in duplicate on CBA5% plates. For NS, the nares of mice were pressed onto a CBA5% plates ten times total (five times/half plate) and exhaled particles were streaked out [1]. On day 3 post-infection, mice were sacrificed and nasal associated lymphoid tissue (NALT; functional homologue to human tonsils) and lungs were removed. Tissues were mechanically homogenised in PBS using the Bullet Blender Homogenizer (Next Advance, USA), serially diluted and dot-plated in duplicate on CBA5% plates. For bacterial enumeration, all plates were incubated at 37 °C overnight and individual haemolytic streptococcal colonies counted. Mice were monitored twice daily until experiment end-point in accordance to established clinical scoring system [2]. Data are represented as the geomean ± geometric SD (cfu/whole tissue) on a Log 10 scale. Source data are provided as a Source Data file.

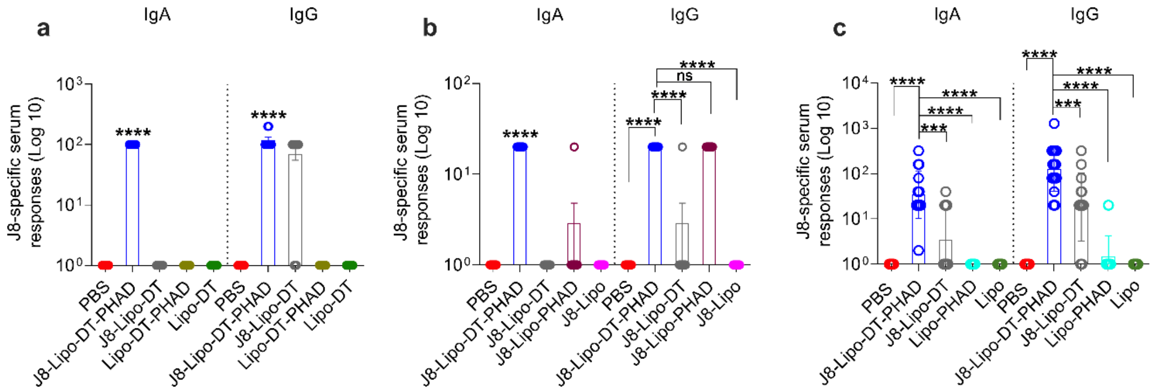

Supplementary Figure 2: Serum IgG and IgA responses for liposomal-based vaccines. Three experiments were conducted to assess the role of vaccine components in immunogenicity and protection. In the first experiment, BALB/c mice (n=10/group; female, 4-6 weeks) were immunized i.n. with PBS, J8-Lipo-DT-PHAD, J8-Lipo-DT, Lipo-DT-PHAD or Lipo-DT on days 0, 21 and 42 **(a)**. In the second experiment, BALB/c mice (n=10/group; female, 4-6 weeks) were immunized i.n. with PBS, J8-Lipo-DT-PHAD, J8-Lipo-DT, J8-Lipo-PHAD, or J8-Lipo on days 0, 21 and 42 **(b)**. In the third experiment, BALB/c mice (n=10-15/group; female, 4-6 weeks) were immunized i.n. with PBS, J8-Lipo-DT-PHAD, J8-Lipo-DT, Lipo-PHAD or Lipo on days 0, 21 and 42 **(c)**. One week post-last-boost J8-specific serum IgA and IgG responses were measured by ELISA and are represented as mean $\pm$ SEM **(a-c)**. Statistical analysis was performed using a nonparametric, unpaired Mann-Whitney U test (one-tailed) (*p<0.05; ***p<0.001; ****p<0.0001). The data in this figure complements figure 1 of the main manuscript. Source data are provided as a Source Data file.

Summarized overview of multiple URT infections with J8-Lipo-DT-PHAD and J8-Lipo-DT vaccinated mice. We graphed the bacterial burden data from 11 different J8-Lipo-DT-PHAD vaccination-challenge experiments (denoted A-K, Supp. Figure 3a-d), and 4 different J8-Lipo-DT vaccination-challenge experiments (denoted A-D, Supp. Figure 3e-h). Some of the individual experiments represent the control groups included in other figures (as listed in the Supp. Figure 3 legend). For 10 out of the 11 experiments, mice were challenged 2-weeks post-last-vaccine boost. In the memory experiment (denoted experiment B), mice were challenged 30-weeks post-last-vaccine boost. In all 11 experiments involving a total of 115 mice vaccinated with J8-Lipo-DT-PHAD and 110 control mice, there was a reduction in the geomean bacterial burden in the NALT of between 35% and 99% (Supp. Figure 3a) with a mean cumulative reduction of 88% ($P<0.0001$; Supp. Figure 3b). For the lungs, the geomean percent reduction ranged from 69% to 99% with a mean of 93% ($P<0.0001$; Supp. Figure 3c-d). For the 4 experiments involving J8-Lipo-DT, similar reductions in bioburden were seen in NALT and lung as recorded for the J8-Lipo-DT-PHAD experiments with overall mean reductions of 97% and 89%, respectively ($P<0.0001$; Supp. Figure 3e-h). In all 4 J8-Lipo-DT experiments the mice were challenged 2-weeks post-last-vaccine boost. The percent (%) reduction formula = ((geomean cfu of PBS group – geomean cfu of J8-Lipo-DT-PHAD group)/geomean cfu of PBS group)*100.

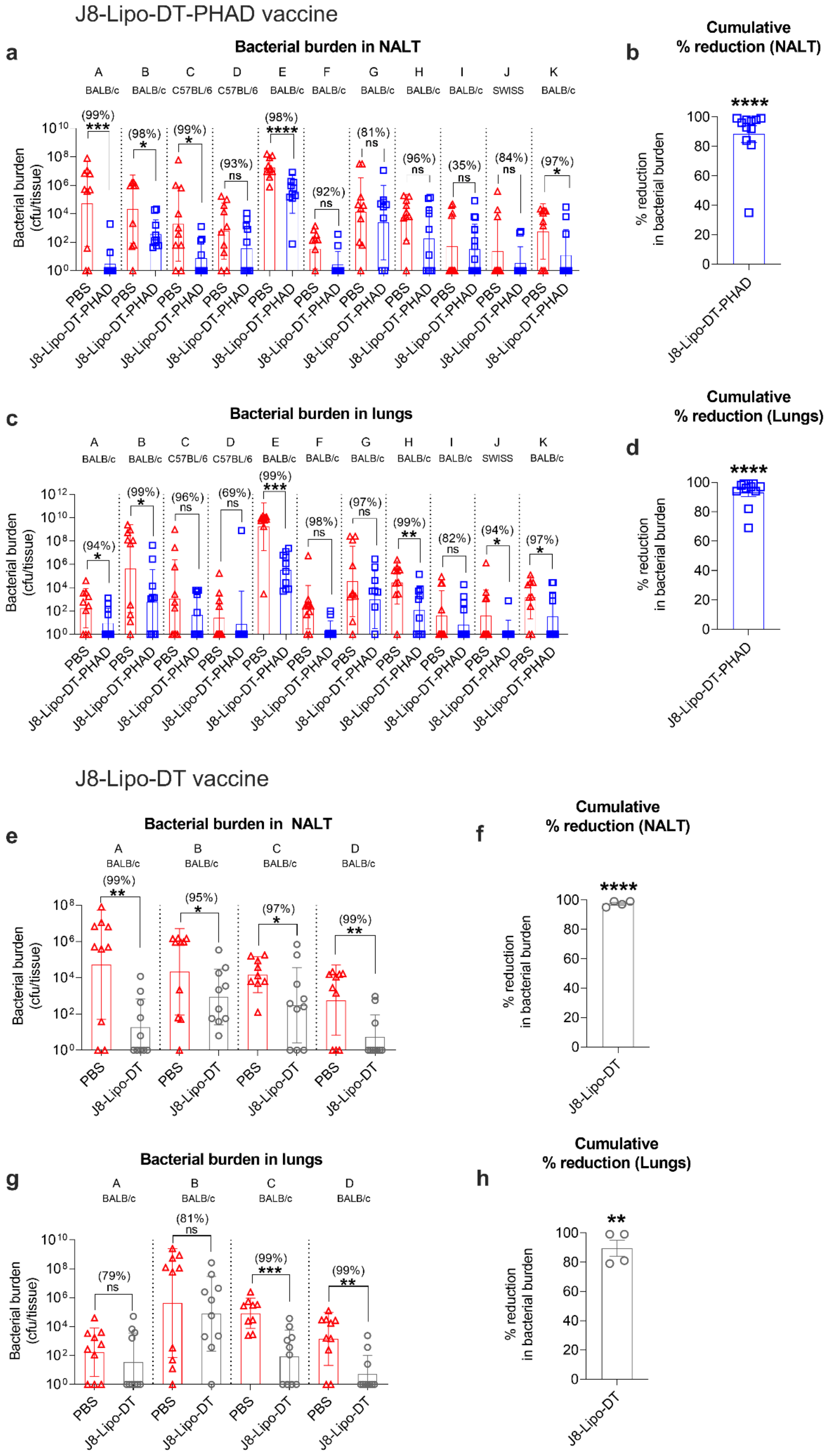
J8-Lipo-DT-PHAD vaccine
a
Bacterial burden in NALT
A B C D E F G H I J K
BALB/c BALB/c C57BL/6 C57BL/6 BALB/c BALB/c BALB/c BALB/c BALB/c SWISS BALB/c
(99%) *** (98%) * (99%) * (93%) ns (98%) **** (92%) ns (81%) ns (96%) ns (35%) ns (84%) ns (97%) *
Bacterial burden (cfu/tissue)
PBS
J8-Lipo-DT-PHAD
b
Cumulative % reduction (NALT)
****
% reduction in bacterial burden
J8-Lipo-DT-PHAD
c
Bacterial burden in lungs
A B C D E F G H I J K
BALB/c BALB/c C57BL/6 C57BL/6 BALB/c BALB/c BALB/c BALB/c BALB/c SWISS BALB/c
(94%) * (99%) * (96%) ns (69%) ns (99%) *** (98%) ns (97%) ns (99%) ** (82%) ns (94%) * (97%) *
Bacterial burden (cfu/tissue)
PBS
J8-Lipo-DT-PHAD
d
Cumulative % reduction (Lungs)
****
% reduction in bacterial burden
J8-Lipo-DT-PHAD
J8-Lipo-DT vaccine
e
Bacterial burden in NALT
A B C D
BALB/c BALB/c BALB/c BALB/c
(99%) ** (95%) * (97%) * (99%) **
Bacterial burden (cfu/tissue)
PBS
J8-Lipo-DT
f
Cumulative % reduction (NALT)
****
% reduction in bacterial burden
J8-Lipo-DT
g
Bacterial burden in lungs
A B C D
BALB/c BALB/c BALB/c BALB/c
(79%) ns (81%) ns (99%) *** (99%) **
Bacterial burden (cfu/tissue)
PBS
J8-Lipo-DT
h
Cumulative % reduction (Lungs)
**
% reduction in bacterial burden
J8-Lipo-DT

Supplementary Figure 3: Summarized overview of multiple URT infections with J8-Lipo-DT-PHAD and J8-Lipo-DT vaccinated mice. Data represent bacterial burden enumeration in NALT and lungs from 11 different URT infection experiments with J8-Lipo-DT-PHAD (denoted a-k) and 4 different URT infection experiments with J8-Lipo-DT vaccination (denoted a-d). The geomean percent reduction for each experiment is presented on top. Bacterial burden in NALT for each individual experiment (J8-Lipo-DT-PHAD) **(a)**, cumulative percent reduction in NALT (J8-lipo-DT-PHAD) **(b)**, bacterial burden in lungs (J8-Lipo-DT-PHAD) **(c)**, cumulative percent reduction in lungs (J8-Lipo-DT-PHAD) **(d)**, bacterial burden in NALT (J8-Lipo-DT) **(e)**, cumulative percent reduction in NALT (J8-lipo-DT) **(f)**, bacterial burden in lungs (J8-Lipo-DT) **(g)** and cumulative percent reduction in lungs (J8-Lipo-DT) **(h)**. Some of the data here are also presented elsewhere in the manuscript. For the J8-Lipo-DT-PHAD experiments, data from experiment A is represented in 1d and experiment B in 3g (experiments C-K have not been represented in this manuscript). For the J8-Lipo-DT experiments, data from experiment A is represented in 1d and experiment B in 3g (experiments C-D have not been represented in this manuscript). Data are represented as geomean $\pm$ geometric SD (cfu/tissue) on a Log 10 scale. Data from different mouse strains are shown to present the adaptability of the URT challenge model in mice with different MHC haplotypes. Statistical analysis was performed using a nonparametric, unpaired Mann-Whitney U test on the raw CFU values of the test groups (one-tailed – ns $p>0.05$; *$p\leq0.05$; **$p<0.01$; ***$p<0.001$; ****$p<0.0001$). The percent reduction in bacterial burden was calculated by comparing the geomean (cfu/tissue) of the PBS group with the geomean (cfu/tissue) of the vaccine group. Source data are provided as a Source Data file.

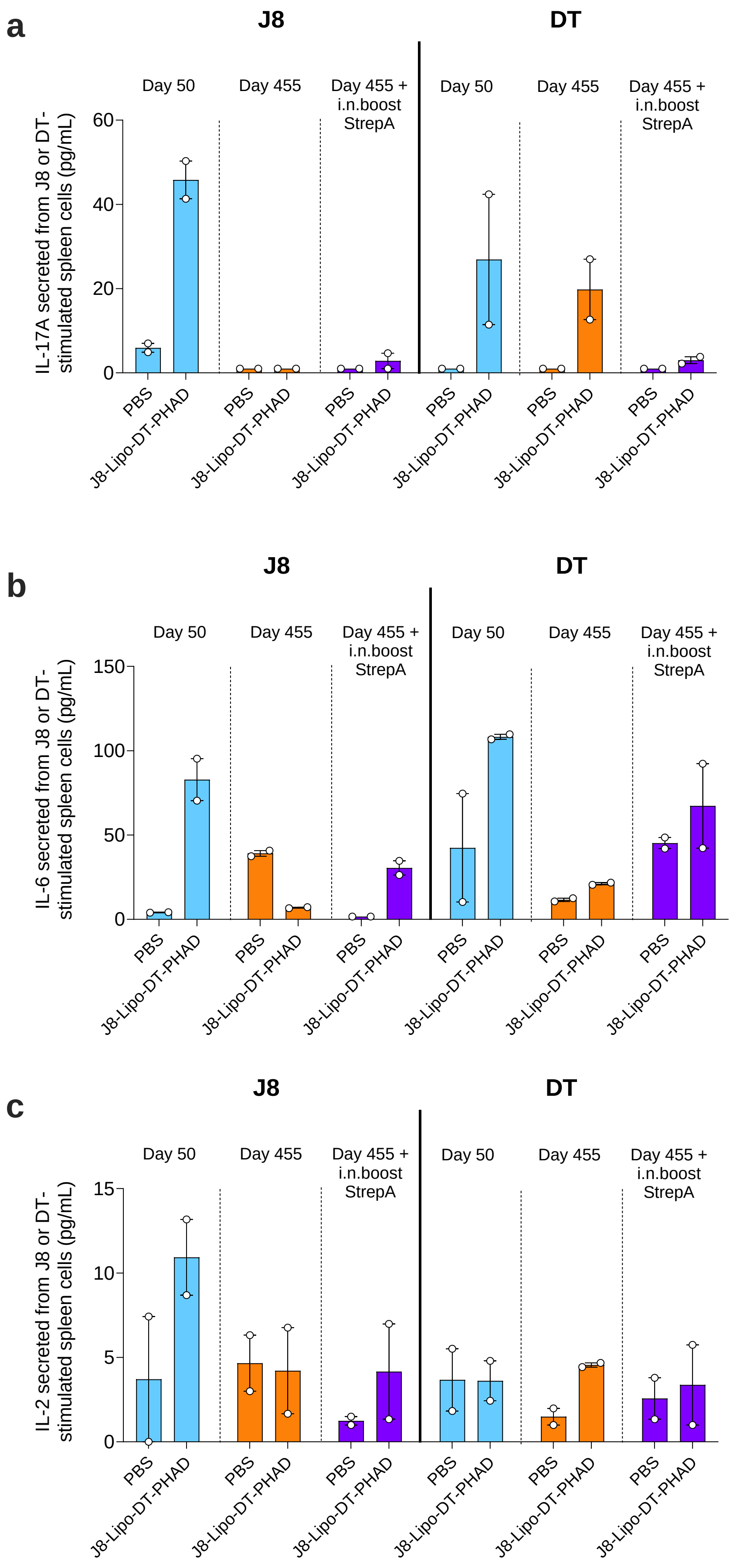

a
J8
DT
Day 50
Day 455
Day 455 + i.n.boost StrepA
IL-17A secreted from J8 or DT-stimulated spleen cells (pg/mL)
0
20
40
60
PBS
J8-Lipo-DT-PHAD
b
IL-6 secreted from J8 or DT-stimulated spleen cells (pg/mL)
0
50
100
150
c
IL-2 secreted from J8 or DT-stimulated spleen cells (pg/mL)
0
5
10
15

Supplementary Figure 4: Longevity of cytokine responses following vaccination with J8-Lipo-DT-PHAD. Spleens were excised and splenocytes were stimulated *ex-vivo* with J8 or DT (50 µg/well) for 72 h and supernatants collected to measure cytokine levels via cytometric bead array. Concentration (pg/mL) of secreted IL-17A **(a)**, IL-6 **(b)** and IL2 **(c)** in PBS or J8-Lipo-DT-PHAD vaccinated mice on day 50, day 455 and day 455 + i.n. boost with StrepA (*S. pyogenes* 2031). The data are normalized to the media stimulation control for each group. Data are represented as mean ± SEM. Statistical analysis was performed using a parametric, unpaired Mann-Whitney U test (one-tailed) to compare test groups to the PBS control group (ns – not significant). The data in this figure complements the longevity cytokine data in figure 3 of the main manuscript. Source data are provided as a Source Data file.

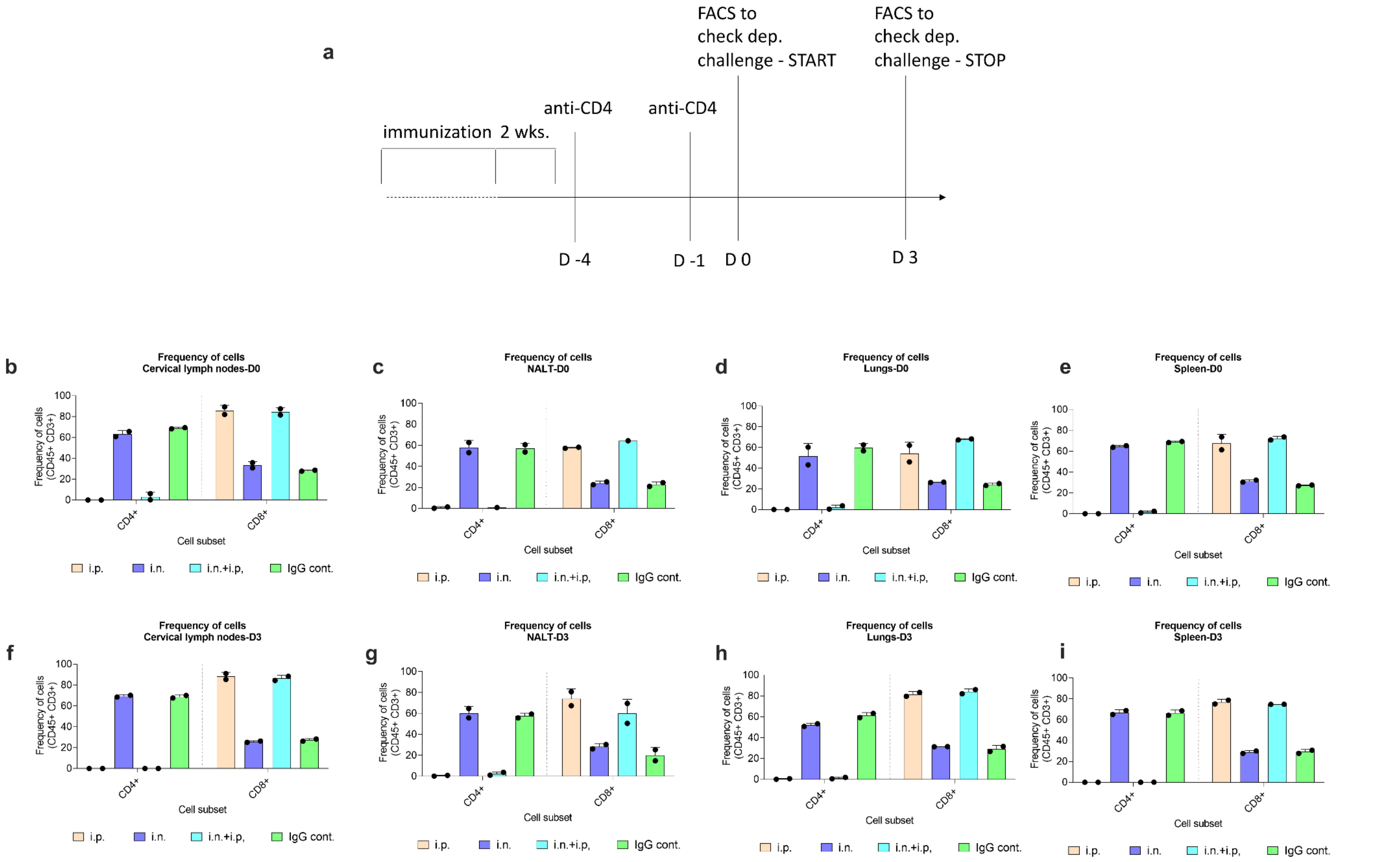
a
FACS to check dep. challenge - START
FACS to check dep. challenge - STOP
anti-CD4
anti-CD4
immunization 2 wks.
D -4
D -1
D 0
D 3
b
Frequency of cells
Cervical lymph nodes-D0
c
Frequency of cells
NALT-D0
d
Frequency of cells
Lungs-D0
e
Frequency of cells
Spleen-D0
f
Frequency of cells
Cervical lymph nodes-D3
g
Frequency of cells
NALT-D3
h
Frequency of cells
Lungs-D3
i
Frequency of cells
Spleen-D3
Frequency of cells (CD45+ CD3+)
CD4+
CD8+
Cell subset
i.p.
i.n.
i.n.+i.p,
IgG cont.

Supplementary Figure 5: Optimizing route of administration and time course of anti-mouse $CD4^+$ mAb (GK1.5) delivery for $CD4^+$ depletion. BALB/c mice (n=4/group; female, 14 weeks old) were treated with 500 µL (215 µg/mouse) of anti-mouse $CD4^+$ mAb intraperitoneally (i.p.), intranasally (i.n.) or i.p. + i.n. on days -4 and -1 before infection (day 0) to achieve >99% depletion of $CD4^+$ cells in tissues. Rat IgG was administered i.p. + i.n. and used as a negative control for the experiment **(a).** $CD4^+$ depletion was confirmed on days 0 and 3 post-mAb administration. Cervical lymph nodes, NALT, lungs and spleen were excised and stained with a cocktail of live/Dead NIR, CD45 BUV395, CD3 PE-CF594, CD4 (RM4) BV510 and CD8a PECy7. $CD4^+$ cells are represented as $CD45^+CD3^+CD4^+$ and $CD8^+$ cells are represented as $CD45^+CD3^+CD8^+$. For day 0 post-depletion, data are represented as frequency of $CD4^+$ and $CD8^+$ cells for- cervical lymph nodes **(b)**, NALT **(c)**, lungs **(d)** and spleen **(e)**. For day 3 post-depletion, data are represented as frequency of $CD4^+$ and $CD8^+$ cells $\pm$ SD for - cervical lymph nodes **(f)**, NALT **(g)**, lungs **(h)** and spleen **(i)**. Samples were run on a BD LSRFortessa cytometer and data analyzed with FlowJo v10.8 software (BD Biosciences). Source data are provided as a Source Data file.

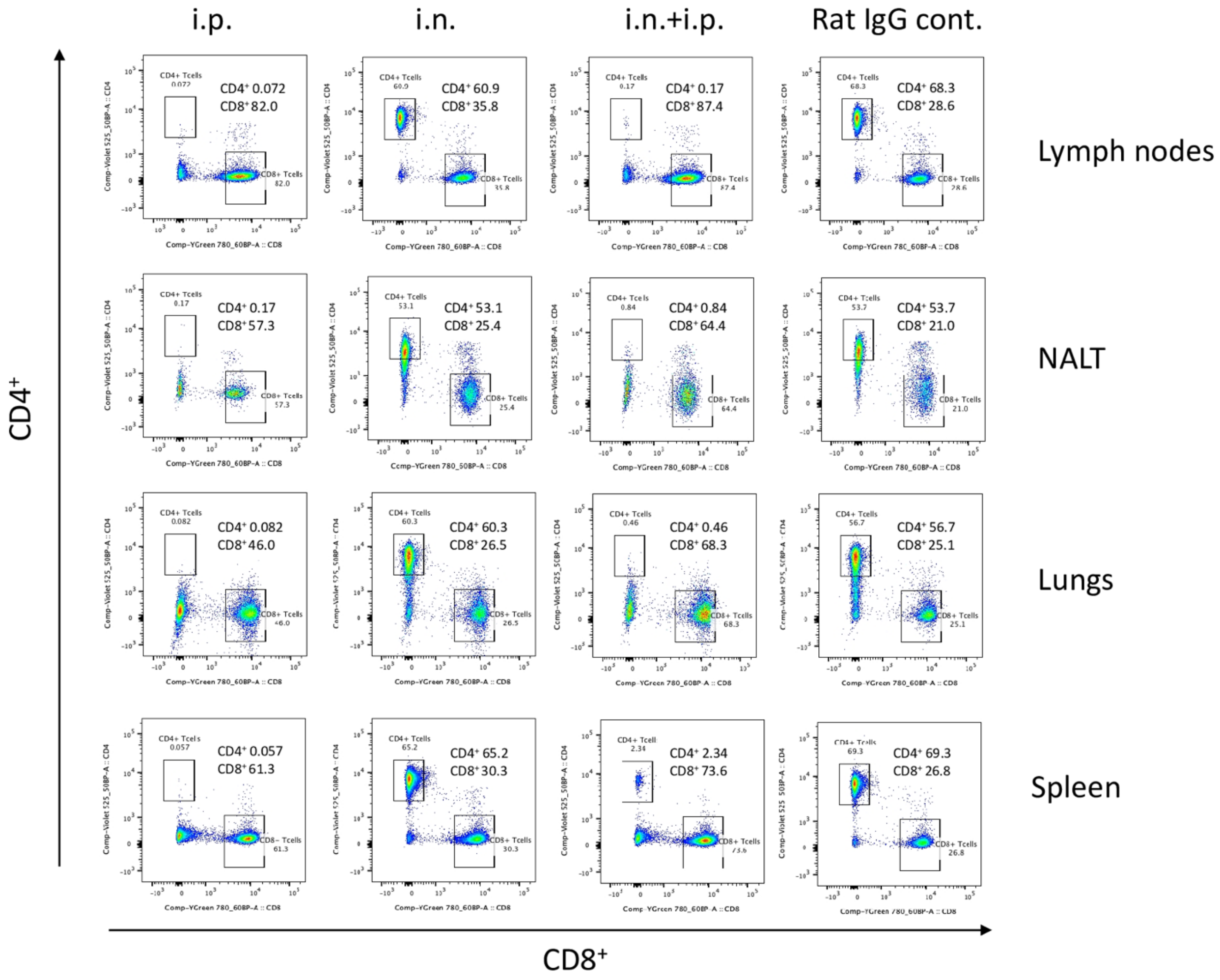

Supplementary Figure 6: Optimizing route of administration and time course of anti-mouse CD4$^+$ mAb (GK1.5) delivery for CD4$^+$ depletion (flow cytometry plots). BALB/c were treated with anti-mouse CD4$^+$or rat IgG (as above; Supp. Figure 5). Data are representative of CD4$^+$ depletion on day 3 post GK 1.5 mAb administration in cervical lymph nodes, NALT, lungs and spleen. Cervical lymph nodes, NALT, lungs and spleen were excised and stained with a cocktail of live/Dead NIR, CD45 BUV395, CD3 PE-CF594, CD4 (RM4) BV510 and CD8a PECy7. Frequency of CD4$^+$ T cells (represented as CD45$^+$CD3$^+$CD4$^+$) and CD8$^+$ T-cells (represented as CD45$^+$CD3$^+$CD8$^+$) on day 3 post-mAb administration is represented within each plot. Samples were run on a BD LSRFortessa cytometer and data analyzed with FlowJo v10.8 software (BD Biosciences).

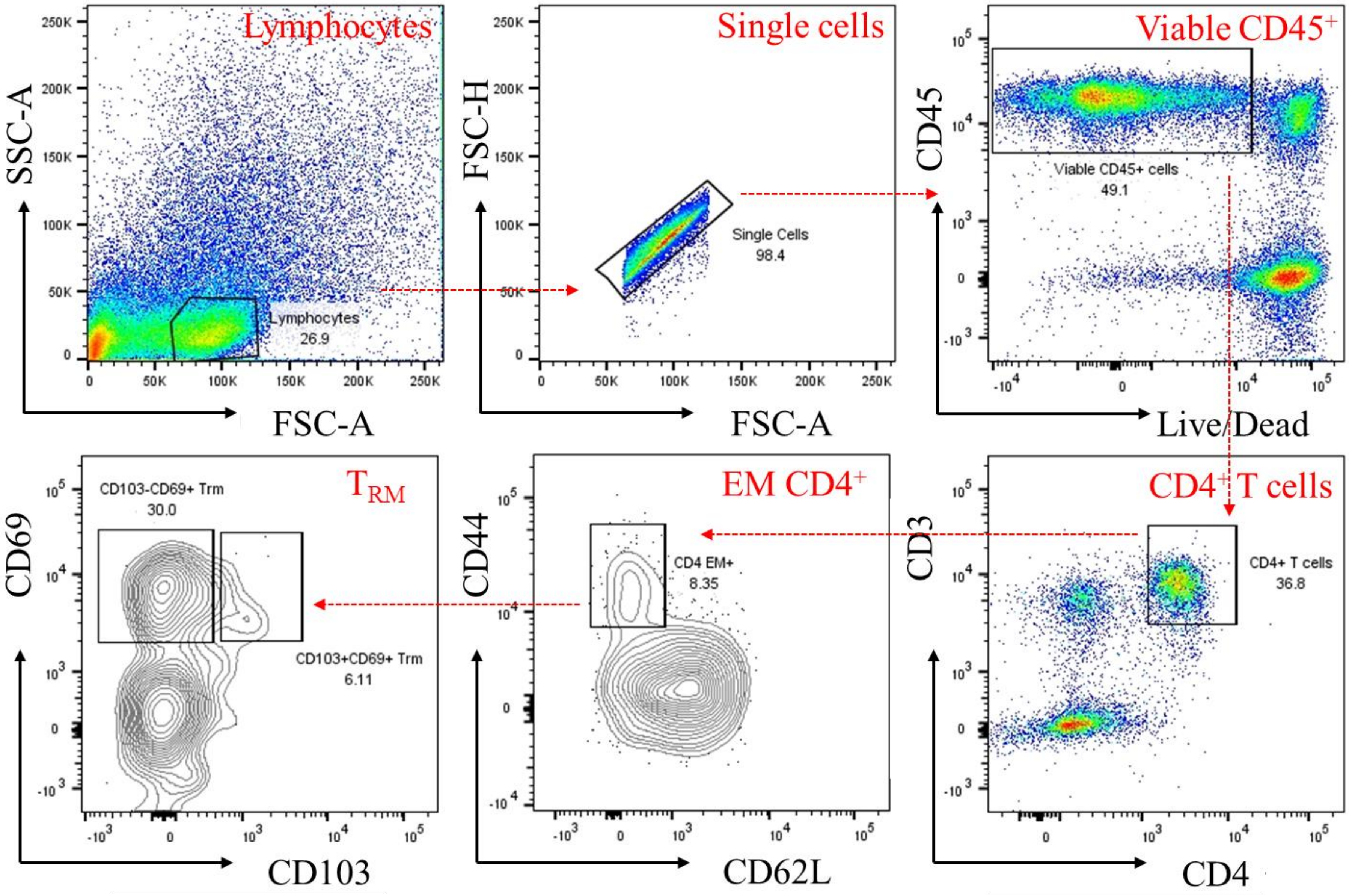

Lymphocytes
Lymphocytes 26.9
SSC-A
FSC-A
Single cells
Single Cells 98.4
FSC-H
FSC-A
Viable CD45+
Viable CD45+ cells 49.1
CD45
Live/Dead
CD4+ T cells
CD4+ T cells 36.8
CD3
CD4
EM CD4+
CD4 EM+ 8.35
CD44
CD62L
TRM
CD103-CD69+ Trm 30.0
CD103+CD69+ Trm 6.11
CD69
CD103

Supplementary Figure 7: Gating strategy for identification of $CD4^{+}T_{RM}$ cells ($CD44^{+}CD62L^{-}CD103^{\pm}CD69^{+}$) in lungs. Representative image of flow cytometry staining of lung cells isolated from a mouse vaccinated with J8-Lipo-DT-PHAD. Samples were run on a BD LSRFortessa cytometer and data analyzed with FlowJo v10.8 software (BD Biosciences).

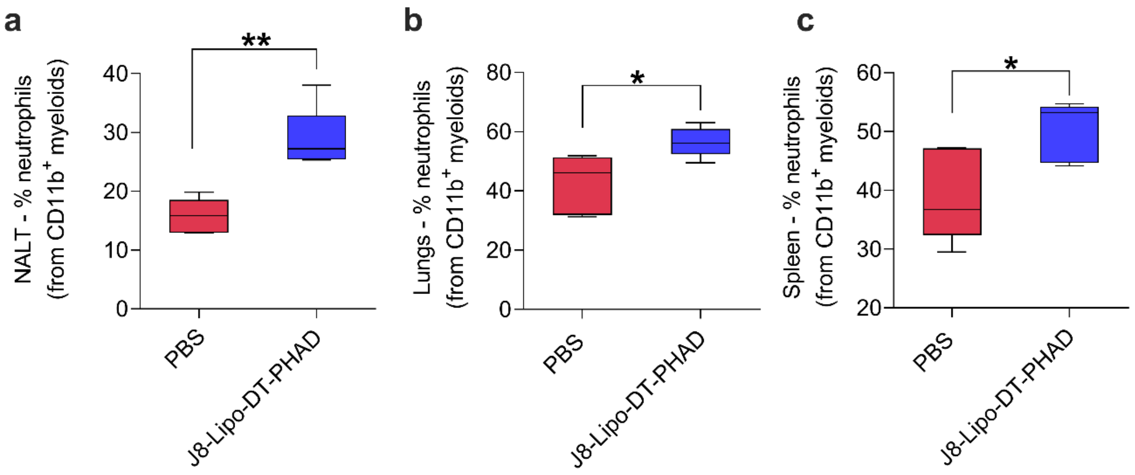

Supplementary Figure 8: Recruitment of neutrophils in vaccinated NALT, lungs and spleen. Neutrophils in the NALT **(a)**, lungs **(b)** and spleen **(c)** of J8-Lipo-DT-PHAD vaccinated and PBS (control) mice were characterized by $CD45^{+}CD11b^{+}Ly6G^{+}$ cells (n=5/group, female). Data are represented as boxplot (min/max; medium with SEM). Statistical analysis was performed using a nonparametric, unpaired Mann-Whitney U test (one-tailed) (*p<0.05; **p<0.01). Samples were run on a BD LSRFortessa cytometer and data analyzed with FlowJo v10.8 software (BD Biosciences). Source data are provided as a Source Data file.

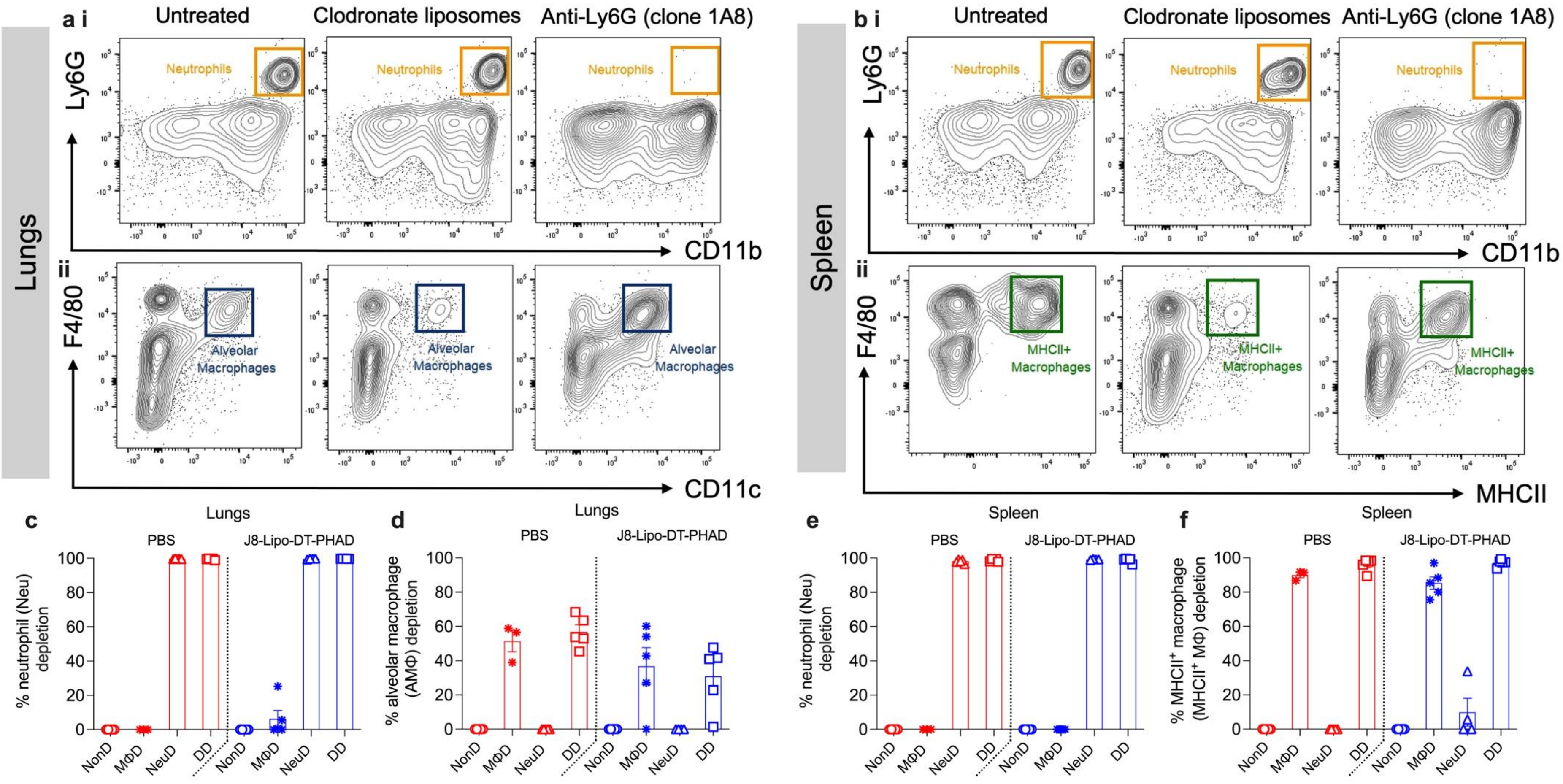

a i
ii
Lungs
Untreated
Clodronate liposomes
Anti-Ly6G (clone 1A8)
Ly6G
CD11b
F4/80
CD11c
Neutrophils
Alveolar Macrophages
b i
ii
Spleen
MHCII
MHCII+ Macrophages
c
Lungs
PBS
J8-Lipo-DT-PHAD
% neutrophil (Neu) depletion
d
% alveolar macrophage (AMΦ) depletion
e
Spleen
f
% MHCII+ macrophage (MHCII+ MΦ) depletion
NonD
MΦD
NeuD
DD

Supplementary Figure 9: Characterizing neutrophil and macrophage depletion in the lungs and spleen. BALB/c mice (n=5/group; female, 4-6 weeks old) were immunized with PBS or J8-Lipo-DT-PHAD. Two weeks post-last-immunization mice were depleted of neutrophils (NeuD), macrophages (MΦD), double depleted (DD; neutrophils + macrophages) or left non-depleted (NonD). For neutrophil depletion, mice were treated with 0.5 mg /mouse (100 μL) of anti-Ly6G mAb (clone 1A8; BioXcell) on days 3, -2 and -1 relative to challenge on day 0. For macrophage depletion, mice were treated with 100 μL (0.5 mg/mouse) of ClLip i.v. and 50 μL (0.25 mg/mouse) of ClLip (5 mg/mL) i.n. on days -3, -2, -1 and + 1 relative to challenge on day 0. For double depletion, BALB/c mice (n=3-5/group; female, 11-12 weeks old) were treated with anti-Ly6G mAb + ClLip on days -3, -2 and -1 and with ClLip on day + 1 relative to challenge on day 0. Neutrophil depletion in the lungs **(a i)** and spleen **(b i)** were characterized by $CD45^+CD11b^+Ly6G^+$ cells. Alveolar macrophage depletion in the lungs **(a ii)** were characterized by $CD45^+$ $CD11c^+$ $F4/80^+$ cells and macrophages in spleen **(b ii)** were characterized by $CD45^+CD11b^+CD24^-MHCII^+F4/80^+$ cells. Samples were run on LSRFortessa cytometer. Figures **a-b** represent flow cytometry contour plots analysed with FlowJo v10.8 software (BD Biosciences) software. Percent neutrophil and macrophage depletion in the lungs and spleen were calculated in comparison to non-depleted controls. Percent neutrophil depletion in the lungs **(c)**, percent alveolar macrophage depletion in the lungs **(d)**, percent neutrophil depletion in the spleen **(e)** and percent macrophage depletion in the spleen **(f)**. Data are presented as mean ± SEM. Source data are provided as a Source Data file.

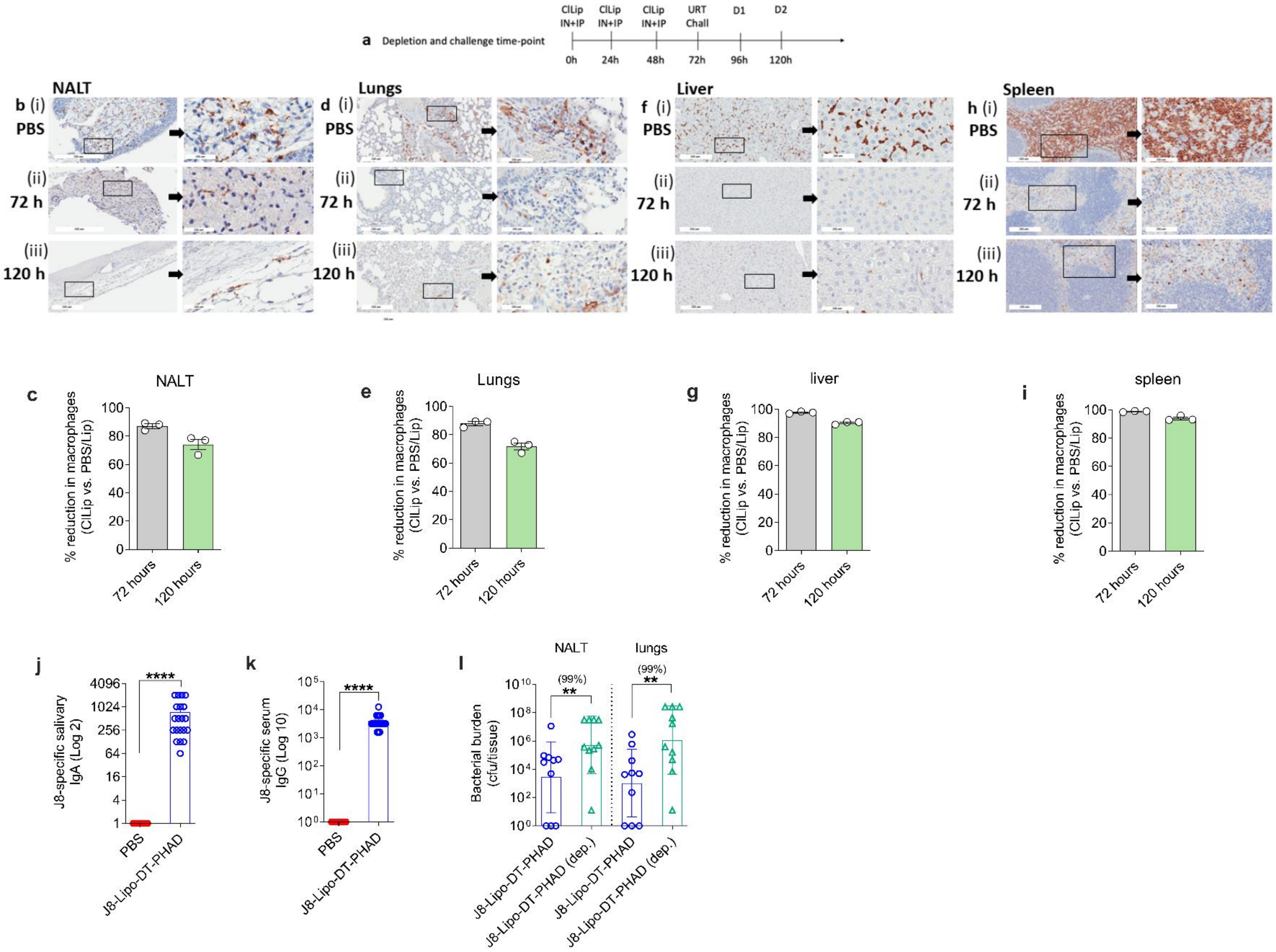
a Depletion and challenge time-point
ClLip IN+IP
ClLip IN+IP
ClLip IN+IP
URT Chall
D1
D2
0h
24h
48h
72h
96h
120h
NALT
b (i) PBS
(ii) 72 h
(iii) 120 h
Lungs
d (i) PBS
(ii) 72 h
(iii) 120 h
Liver
f (i) PBS
(ii) 72 h
(iii) 120 h
Spleen
h (i) PBS
(ii) 72 h
(iii) 120 h
c
NALT
e
Lungs
g
liver
i
spleen
% reduction in macrophages (ClLip vs. PBS/Lip)
72 hours
120 hours
j
J8-specific salivary IgA (Log 2)
PBS
J8-Lipo-DT-PHAD
k
J8-specific serum IgG (Log 10)
l
NALT
lungs
(99%)
Bacterial burden (cfu/tissue)
J8-Lipo-DT-PHAD
J8-Lipo-DT-PHAD (dep.)

Supplementary Figure 10: Role of macrophages in J8-Lipo-DT-PHAD-mediated protection. Dose and time course optimization of Clodronate liposome (ClLip) delivery for macrophage depletion.BALB/c mice (n=5/group; female, 11-12 weeks old) were treated with 100 µL (0.5 mg/mouse) of ClLip i.p. and 50µL (0.25 mg/mouse) of ClLip (5 mg/mL) i.n. on days -3, -2 and -1 before challenge (day 0) to achieve >70-90% depletion of macrophages in tissues **(a)**. Macrophage depletion in NALT **(b-c)**, lungs **(d-e)**, liver **(f-g)** and **(h-i)** spleen biopsy samples were assessed via histopathology. Sections were scanned and read at high magnification using ImageScope software. F4/80+ cells were counted at 20x magnification in 5 high-powered fields and expressed as the average number of positive cells/high-powered field. **(i-iii)** A F4/80-stained tissue section from a mouse treated with **(i)** PBS/Liposomes (control), **(ii)** ClLip, at 72 h post-treatment (day of URT-infection) and **(iii)** ClLip at 120 h post-treatment (day of tissue collection for bacterial burden enumeration). **(j-l) J8-Lipo-DT-PHAD-mediated protection following macrophage depletion.** BALB/c mice (n=10/group; female, 4-6 weeks old) were immunized i.n. with J8-Lipo-DT PHAD. One week post-last-boost J8-specific salivary IgA **(j)** and serum IgG **(k)** antibody responses were measured by ELISA and are represented as mean ± SEM. Two weeks after last boost mice were administered an optimized dose of ClLip (0.5 mg/mouse i.p. and 0.25 mg/mouse i.n.) on days -3, -2 and -1 before challenge (day 0) to achieve >80% depletion of macrophages in all tissues tested. Mice were challenged via the URT with S. pyogenes 2031 ($5x10^6$ cfu/mouse). On day 2 post-challenge all surviving mice were euthanized and NALT and lungs collected to determine *S. pyogenes* burden **(l)**. Data represent the geomean ± geometric SD (cfu/tissue) on a Log 10 scale. Statistical analysis was performed using a nonparametric, unpaired Mann-Whitney U test (one-tailed) (**p<0.01; ****p<0.0001). Percent reduction was calculated in comparison to the geomean of the control cohort. Source data are provided as a Source Data file.

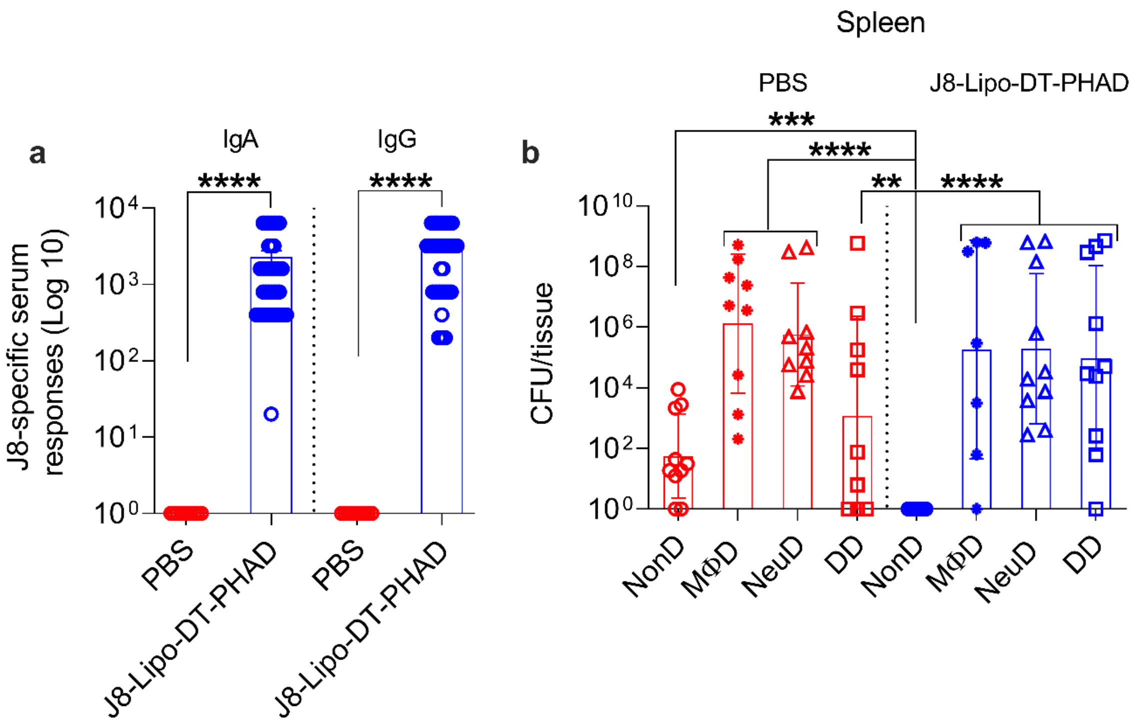

Supplementary Figure 11: J8-Lipo-DT-PHAD-mediated systemic protection following depletion. BALB/c mice (n=10/group; female, 4-6 weeks old) were immunized i.n. with J8-Lipo-DT PHAD or PBS. One week post-last-boost J8-specific serum IgA and IgG **(a)** antibody responses were measured by ELISA and are represented as mean + SEM. Two weeks after last boost mice were depleted of neutrophils, macrophages, double depleted or left non-depleted as per protocol in figure 7 of the main manuscript. Mice were challenged via the URT with *S. pyogenes* 2031 ($5x10^6$ cfu/mouse). On day 2 post-challenge (challenge time-point day 0) all surviving mice were euthanized and spleens excised to determine *S. pyogenes* burden **(b)**. Data are represented as geomean + geometric SD (cfu/tissue) on a Log 10 scale. Statistical analysis was performed using a nonparametric, unpaired Mann-Whitney U test on the raw CFU values (one-tailed) (***p<0.001; ****p<0.0001). The data in this figure complements figure 8 of the main manuscript. Source data are provided as a Source Data file.

Supplementary Table 1: List of antibodies used in flow cytometry

Antibody	Clone	Fluorophore	Company	Dilution
CD45	30-F11	BUV395	BD Biosciences	1:500
CD3	145-2C11	PE/DAZZLE 594	BD Biosciences	1:400
CD4	RM4-5	BV510	BD Biosciences	1:400
CD8a	53-6.7	PECy7	Invitrogen	1:400
F4/80	T45-2342	PE	BD Biosciences	1:400
CD11b	M1/70	BUV737	BD Biosciences	1:400
CD11c	N418	AF488	BioLegend	1:400
CD24	M1/69	BV510	BD Biosciences	1:300
MHC11 (1A/1E)	M5/114.15.2	AF700	BioLegend	1:400
Ly6G	1A8	BV510	BioLegend	1:300
CCR6 (CD196)	29-2L17	PE	BioLegend	1:200
CD62L	MEL-14	PerCP-Cy5.5	BD Biosciences	1:300
CD44	IM7	BV785	BioLegend	1:300
CD69	H1.2F3	BV421	BioLegend	1:100
CD103	M290	BV711	BD Biosciences	1:100
IL17-A	TC11-18H10.1	APC	BioLegend	1:100
Live/Dead	Dead cell stain	Near-IR	Invitrogen	1:400

Supplementary References

1. Alam FM, Turner CE, Smith K, Wiles S, Sriskandan S. Inactivation of the CovR/S virulence regulator impairs infection in an improved murine model of Streptococcus pyogenes naso-pharyngeal infection. *PLoS One* **8**, e61655 (2013).

2. Pandey M, Good MF. A Superficial Skin Scarification Method in Mice to Mimic Streptococcus pyogenes Skin Infection in Humans. *Methods Mol Biol* **2136**, 287-301 (2020).
