# Supplementary material for: A Glycolipidated-liposomal peptide vaccine confers long-term mucosal protection against Streptococcus pyogenes via IL-17, macrophages and neutrophils
Source: Nat Commun. 2023 Sep 25;14:5963. doi: 10.1038/s41467-023-41410-7 (PMC10520070; doi:10.1038/s41467-023-41410-7)
Supplement: Supplementary file 3 — Reporting Summary [file 41467_2023_41410_MOESM3_ESM.pdf]

nature portfolio

Corresponding author(s): Assoc. Prof. Manisha Pandey and Prof. Michael Good

Last updated by author(s): 28 July 2023

Reporting Summary

Nature Portfolio wishes to improve the reproducibility of the work that we publish. This form provides structure for consistency and transparency in reporting. For further information on Nature Portfolio policies, see our Editorial Policies and the Editorial Policy Checklist.

Please do not complete any field with "not applicable" or n/a. Refer to the help text for what text to use if an item is not relevant to your study.
For final submission: please carefully check your responses for accuracy; you will not be able to make changes later.

Statistics

For all statistical analyses, confirm that the following items are present in the figure legend, table legend, main text, or Methods section.

n/a	Confirmed	
	X	The exact sample size (*n*) for each experimental group/condition, given as a discrete number and unit of measurement
	X	A statement on whether measurements were taken from distinct samples or whether the same sample was measured repeatedly
	X	The statistical test(s) used AND whether they are one- or two-sided<br>*Only common tests should be described solely by name; describe more complex techniques in the Methods section.*
	X	A description of all covariates tested
	X	A description of any assumptions or corrections, such as tests of normality and adjustment for multiple comparisons
	X	A full description of the statistical parameters including central tendency (e.g. means) or other basic estimates (e.g. regression coefficient) AND variation (e.g. standard deviation) or associated estimates of uncertainty (e.g. confidence intervals)
	X	For null hypothesis testing, the test statistic (e.g. *F*, *t*, *r*) with confidence intervals, effect sizes, degrees of freedom and *P* value noted<br>*Give P values as exact values whenever suitable.*
X		For Bayesian analysis, information on the choice of priors and Markov chain Monte Carlo settings
X		For hierarchical and complex designs, identification of the appropriate level for tests and full reporting of outcomes
X		Estimates of effect sizes (e.g. Cohen's *d*, Pearson's *r*), indicating how they were calculated

Our web collection on statistics for biologists contains articles on many of the points above.

Software and code

Policy information about availability of computer code

Data collection	Flow Cytometry: BD LSRFortessa Cell Analyzer; ELISA: Tecan Infinite M200 Pro
Data analysis	Slide viewing: Aperio ImageScope Software v12.3.3; Flow cytometry analyses: FlowJo v10.8 software (BD Biosciences); Cytokine bead array analyses: FCAP Array (v.3.0); Power calculations: G*Power software v3.1.9.7

For manuscripts utilizing custom algorithms or software that are central to the research but not yet described in published literature, software must be made available to editors and reviewers. We strongly encourage code deposition in a community repository (e.g. GitHub). See the Nature Portfolio guidelines for submitting code & software for further information.

Data

Policy information about availability of data

All manuscripts must include a data availability statement. This statement should provide the following information, where applicable:

- Accession codes, unique identifiers, or web links for publicly available datasets
- A description of any restrictions on data availability
- For clinical datasets or third party data, please ensure that the statement adheres to our policy

All data necessary to interpret the findings and draw conclusions of the study are included within the manuscript and the supplementary information. This statement has been included under 'Data availability statement' in the methods section of the manuscript.

Research involving human participants, their data, or biological material

Policy information about studies with human participants or human data. See also policy information about sex, gender (identity/presentation), and sexual orientation and race, ethnicity and racism.

Reporting on sex and gender	This research involves no human participants.
Reporting on race, ethnicity, or other socially relevant groupings	N/A
Population characteristics	N/A
Recruitment	N/A
Ethics oversight	N/A

Note that full information on the approval of the study protocol must also be provided in the manuscript.

Field-specific reporting

Please select the one below that is the best fit for your research. If you are not sure, read the appropriate sections before making your selection.

[X] Life sciences   [ ] Behavioural & social sciences   [ ] Ecological, evolutionary & environmental sciences

For a reference copy of the document with all sections, see nature.com/documents/nr-reporting-summary-flat.pdf

Life sciences study design

All studies must disclose on these points even when the disclosure is negative.

Sample size

Vaccine immunogenicity/efficacy experiments - Figures 1c-h, 3b-e, 4a-b, 5a-c, 6a-f and 8a-b, we used n= 10 mice/group. This is based on a Wilcoxon-Mann-Whitney t-test (for non-normal data) completed with a priori power analysis to compute the required sample size. A Shapiro-Wilk test was used to calculate that the data was not normally distributed. The parent distribution input parameter for the power analysis was logistic due to the heavy tails on the distribution. Based on the data from 3 separate upper respiratory tract (URT) challenge experiments and the following assumptions: power of 0.8, alpha value of 0.05, two sided (to test both if the mean is significantly greater than x and if the mean significantly less than x), and not normally distributed - an average group number of 10-15 will provide us with a medium effect size and a power of 0.8 to determine biological differences within a 95% confidence interval (CI).

For all vaccine immunogenicity/efficacy experiments, data are represented as geomean +/- geometric SD (cfu/tissue) on a Log 10 scale. Statistical analysis was performed using a nonparametric, unpaired Mann-Whitney U test (one-tailed - ns p>0.05; *p<0.05; **p<0.01; ***p<0.001; ****p<0.0001). Percent reduction in bacterial burden was calculated by comparing the geomean (cfu/tissue) of the group of interest with the geomean (cfu/tissue) of the test group.

For *in-vitro* cell culture experiments - Figures 2a-c, 3j-l, 5d-f, 7a-b and 8c, we used 3-5 mice/group. Based on the data from 3 separate in-vitro experiments and the above assumptions - an average group number of 3 mice will provide us with a medium effect size and a power of 0.8 to determine biological differences within a 95% confidence interval (CI).

For all *in-vitro* cell culture experiments, data are represented mean+/- SEM. Statistical analysis was performed using a nonparametric, unpaired Mann-Whitney U test (one-tailed) or two-way ANOVA, corrected for multiple comparisons using the Sidak's multiple comparison test (ns p>0.05; *p<0.05; **p<0.01; ***p<0.001; ****p<0.0001).

All sample size measurements were calculated using the software G*Power. ARRIVE guidelines were followed for sample size calculations: Percie du Sert N, Hurst V, Ahluwalia A, Alam S, Avey MT, Baker M, et al. (2020) The ARRIVE guidelines 2.0: Updated guidelines for reporting animal research. PloS Biol 18(7): e3000410.

Data exclusions

There were no data exclusions from the analyses.

Replication

Supplementary Figure 3 shows a summarized of multiple URT infections with J8-Lipo-DT-PHAD and J8-Lipo-DT. In this figure we have shown data from 11 different vaccination experiments with J8-Lipo-DT-PHAD (A-D, n= l0-15 mice/group) and four different experiments with J8-Lipo-DT (E-H, n= 10-15 mice/group). Some of the data in this figure represent the control groups in other figures in the manuscript (this is outlined in the figure legend). Supplementary Figure 3 is a transparent overview of all the experiments (replications) we have conducted with the vaccine formulations J8-Lipo-DT-PHAD and J8-Lipo-DT.

Randomization	For all mice experiments, the mice are randomized into cohorts of five by the animal house staff.
Blinding	For vaccine efficacy experiments, mice were immunized intranasally on days 0, 21 and 42. Before bacterial challenge, mice undergoing procedure were blinded by a researcher from the lab not directly involved in the experiment or by the animal house staff. Mice were unblinded once all data had been procured and analyzed.

Behavioural & social sciences study design

All studies must disclose on these points even when the disclosure is negative.

Study description	N/A
Research sample	N/A
Sampling strategy	N/A
Data collection	N/A
Timing	N/A
Data exclusions	N/A
Non-participation	N/A
Randomization	N/A

Ecological, evolutionary & environmental sciences study design

All studies must disclose on these points even when the disclosure is negative.

Study description	N/A
Research sample	N/A
Sampling strategy	N/A
Data collection	N/A
Timing and spatial scale	N/A
Data exclusions	N/A
Reproducibility	N/A
Randomization	N/A
Blinding	N/A

Did the study involve field work? [ ] Yes [X] No

Field work, collection and transport

Field conditions	N/A
Location	N/A
Access & import/export	N/A
Disturbance	N/A

Reporting for specific materials, systems and methods

We require information from authors about some types of materials, experimental systems and methods used in many studies. Here, indicate whether each material, system or method listed is relevant to your study. If you are not sure if a list item applies to your research, read the appropriate section before selecting a response.

Materials & experimental systems

n/a	Involved in the study	
[ ]	[X]	Antibodies
[X]	[ ]	Eukaryotic cell lines
[X]	[ ]	Palaeontology and archaeology
[ ]	[X]	Animals and other organisms
[X]	[ ]	Clinical data
[X]	[ ]	Dual use research of concern
[X]	[ ]	Plants

Methods

n/a	Involved in the study	
[X]	[ ]	ChIP-seq
[ ]	[X]	Flow cytometry
[X]	[ ]	MRI-based neuroimaging

Antibodies

Antibodies used

Antibodies used in ELISA: Goat anti-Mouse lgA Cross-Adsorbed Secondary Antibody, HRP (Cat no. PAl 74397, Life Technologies, USA) and Goat Anti-Mouse lgG (H + L)-HRP (Cat no. 1706516, Bio-Rad, USA).

Antibodies used in flow cytometry/cell depletion: Rat anti-mouse CD4 mAb (Cat no. BE0003-1-25MG, GK 1.5, Assay Matrix, Australia), live/dead NIR (Cat no. 423105, Biolegend, USA), CD45 BUV395 (Cat no. 564279, clone 30-Fll, BD Biosciences, USA), CD3 PE-CF594 (Cat no. 562286, clone 145-2Cll, BD Biosciences, USA), CD4 BV510 (Cat no. 563106, clone RM4-5, BD Biosciences, USA), CD8a PECy7 (Cat no. 25-0081-82, clone 53-6.7, lnvitrogen, USA), F4/80 PE (Cat no. 565409, clone T45-2342, BD Biosciences, USA), CDllb BUV737 (Cat no. 612801, clone Ml/70, BD Biosciences, USA), CDllc AF488 (Cat no. 117311, clone N418, Biolegend, USA), CD24 BV510 (Cat no. 747717, clone Ml/69, BD Biosciences, USA), MHCII (lA/lE) AF700 (Cat no. 107622, clone M5/114.15.2, Biolegend, USA), Ly6G BV510 (Cat no. 127633, clone 1A8, Biolegend, USA), CCR6 (CD196) PE (Cat no. 129804, clone 29-2L17, Biolegend, USA), CD62L PerCP-Cy5.5 (Cat no. 560513, clone MEL-14, BD Biosciences, USA), CD44 BV785 (Cat no. 103059, clone IM7, Biolegend, USA), CD69 BV421 (Cat no. 104545, clone Hl.2F3, Biolegend, USA), CD103 BV711 (Cat no. 563106, clone M290, BD Biosciences, USA) and IL-17A APC (Cat no. 506916, clone TC11-18H10.l, Biolegend, USA).

The dilution for each antibody is listed in the Methods or Supplementary Table 1 of the manuscript.

Validation

Goat anti-Mouse lgA Cross-Adsorbed Secondary Antibody, HRP: Whole antibody cross adsorption against mouse lgM, lgG1, lgG2a, lgG2b and lgG3. The antibody is affinity purified with well-characterized specificity for mouse immunoglobulins. This antibody will not react with human samples. An optimal dilution of 1:1000 was used for this antibody. https://www.thermofisher.com/antibody/product/Goat-anti-Mouse-IgA-Cross-Adsorbed-Secondary-Antibody-Polyclonal/PA1-74397.

Goat Anti-Mouse lgG (H + L)-HRP: Pass for blotting immunoassay, mouse lgG 3.9 ng. Pass for binding Activity@ 490 nm >/=0.12 U/ min. An optimal dilution of 1:3000 was used for this antibody. https://www.bio-rad.com/en-au/sku/1706516-goat-anti-mouse-igg-h-l-hrp-conjugate?ID=1706516.

InVivoMAb anti-mouse CD4 mAb (GK 1.5): Validated through western blot to confirm that this clone binds to its target antigen. The purity is >95% as determined by SDS-PAGE. The mAb is purified from tissue culture supernatant in an animal free facility (protein G purification). Supplementary Figures 5-6 of the manuscript shows the method used to optimize the route of administration and time course of anti-mouse CD4 mAb delivery to achieve >99% depletion. Rat-IgG2b isotype control was used as a negative control for the experiment. https://bioxcell.com/invivomab-anti-mouse-cd4-be0003-1.

Anti-mouse Ly6G mAb (1A8; Gr-1 clone RB6-8C5): Validated through western blot to confirm that this clone binds to its target antigen. The mAb is useful for in-vivo depletion of Gr-1+ myeloid cells. The mAb has 95% purity. Supplementary Figure 9 in the manuscript shows the characterization of the mAb to deplete neutrophils in the lungs and spleen of mice. https://blog.bxcell.com/anti-mouse-ly6g-ly6c-gr-1-clone-rb6-8c5.

Each lot of the following products are quality control tested by immunofluorescent staining with flow cytometric analysis.

Live/dead NIR (Lot no. B305540): For flow cytometry, the suggested dilution is 1:100-1:1000 for 1-10 million cells. It is recommended that the reagent be titrated for optimal performance for each application, as optimal dosage varies with cell type. We have titrated this antibody and for splenocytes (400 000 cells total) we have used an optimal dilution of 1:400. https://www.thermofisher.com/order/catalog/product/L10119.

CD45 BUV395 (30-F11): We have titrated this antibody and for splenocytes (400 000 cells total) we have used an optimal dilution of 1:500. https://www.bdbiosciences.com/en-au/products/reagents/flow-cytometry-reagents/research-reagents/single-color-antibodies-ruo/buv395-rat-anti-mouse-cd45.564279.

CD3 PE-CF594 (145-2C11): We have titrated this antibody and for splenocytes (400 000 cells total) we have used an optimal dilution of 1:400. https://www.bdbiosciences.com/en-au/products/reagents/flow-cytometry-reagents/research-reagents/single-color-antibodies-ruo/pe-cf594-hamster-anti-mouse-cd3e.562332.

CD4 BV510 (RM4): We have titrated this antibody and for splenocytes (400 000 cells total) we have used an optimal dilution of 1:400. https://www.bdbiosciences.com/en-au/products/reagents/flow-cytometry-reagents/research-reagents/single-color-antibodies-ruo/bv510-rat-anti-mouse-cd4.563106.

CD8a PECy7 (53-6.7): We have titrated this antibody and for splenocytes (400 000 cells total) we have used an optimal dilution of 1:400. https://www.thermofisher.com/antibody/product/CD8a-Antibody-clone-53-6-7-Monoclonal/25-0081-82.

F4/80 PE (T45-2342): We have titrated this antibody and for splenocytes (400 000 cells total) we have used an optimal dilution of 1:400. https://www.bdbiosciences.com/en-au/products/reagents/flow-cytometry-reagents/research-reagents/single-color-antibodies-ruo/pe-rat-anti-mouse-f4-80.565410.

CD11b BUV737 (M1/70): We have titrated this antibody and for splenocytes (400 000 cells total) we have used an optimal dilution of 1:400. https://www.bdbiosciences.com/en-au/products/reagents/flow-cytometry-reagents/research-reagents/single-color-antibodies-ruo/buv737-rat-anti-cd11b.612801.

CD11c AF488 (N418): We have titrated this antibody and for splenocytes (400 000 cells total) we have used an optimal dilution of 1:400. https://www.thermofisher.com/antibody/product/CD11c-Antibody-clone-N418-Monoclonal/53-0114-82.

CD24 BV510 (M1/69): We have titrated this antibody and for splenocytes (400 000 cells total) we have used an optimal dilution of 1:300. https://www.bdbiosciences.com/en-au/products/reagents/flow-cytometry-reagents/research-reagents/single-color-antibodies-ruo/bv510-rat-anti-mouse-cd24.747717.

MHCII (1A/1E) AF700 (M5/114.15.2): We have titrated this antibody and for splenocytes (400 000 cells total) we have used an optimal dilution of 1:400. https://www.biolegend.com/en-us/cell-health/alexa-fluor-700-anti-mouse-i-a-i-e-antibody-3413.

Ly6G BV510 (1A8): We have titrated this antibody and for splenocytes (400 000 cells total) we have used an optimal dilution of 1:300. https://www.biolegend.com/de-de/sean-tuckers-tests/brilliant-violet-510-anti-mouse-ly-6g-antibody-9121?GroupID=BLG7232.

CCR6 (CD196) PE (29-2L17): We have titrated this antibody and for splenocytes (400 000 cells total) we have used an optimal dilution of 1:200. https://www.biolegend.com/en-us/products/pe-anti-mouse-cd196-ccr6-antibody-5220.

CD62L PerCP-CyS.6 (MEL-14): We have titrated this antibody and for splenocytes (400 000 cells total) we have used an optimal dilution of 1:300. https://www.bdbiosciences.com/en-au/products/reagents/flow-cytometry-reagents/research-reagents/single-color-antibodies-ruo/percp-cy-5-5-rat-anti-mouse-cd62l.560513.

CD44 BV785 (IM7): We have titrated this antibody and for splenocytes (400 000 cells total) we have used an optimal dilution of 1:300. https://www.biolegend.com/nl-nl/cell-health/brilliant-violet-785-anti-mouse-human-cd44-antibody-7959.

CD69 BV421 (H1.2F3): We have titrated this antibody and for splenocytes (400 000 cells total) we have used an optimal dilution of 1:100. https://www.biolegend.com/en-gb/cell-health/brilliant-violet-421-anti-mouse-cd69-antibody-7358?GroupID=BLG10536.

CD103 BV711 (M290): We have titrated this antibody and for splenocytes (400 000 cells total) we have used an optimal dilution of 1:100. https://www.bdbiosciences.com/en-au/products/reagents/flow-cytometry-reagents/research-reagents/single-color-antibodies-ruo/bv711-rat-anti-mouse-cd103.564320.

IL-17A APC (TC11-18H10.1): We have titrated this antibody and for splenocytes (400 000 cells total) we have used an optimal dilution of 1:100. https://www.biolegend.com/nl-be/explore-new-products/apc-anti-mouse-il-17a-antibody-3540?GroupID=GROUP24.

Eukaryotic cell lines

Policy information about cell lines and Sex and Gender in Research

Cell line source(s)	N/A
Authentication	N/A
Mycoplasma contamination	N/A
Commonly misidentified lines (See ICLAC register)	N/A

Palaeontology and Archaeology

Specimen provenance	N/A
Specimen deposition	N/A
Dating methods	N/A

☐ Tick this box to confirm that the raw and calibrated dates are available in the paper or in Supplementary Information.

Ethics oversight	N/A

Note that full information on the approval of the study protocol must also be provided in the manuscript.

Animals and other research organisms

Policy information about studies involving animals; ARRIVE guidelines recommended for reporting animal research, and Sex and Gender in Research

Laboratory animals

BALB/c and C57BL/6 mice female 4-6 weeks of age were sourced from the Animal Resource Centre (ARC), Western Australia, Australia.

plgR knock-out (plgR -/-; C57BL/6 background), B-cell knock-out (MUMT; C57BL/6 background) and IL-17 knock-out (IL-17-/-; BALB/c background) were bred in-house at the Griffith University Animal Facility. plgR-/- and MUMT mice were acquired from Queensland Institute for Medical Research Berghofer. IL-17-/- mice were acquired from Yoichiro Iwakura (Tokyo University of Science, Japan).

All mice were housed in a PC2 certified animal facility in individually ventilated cages (IVC) with a maximum of 5 mice/cage. The relative humidity ranged between 45-65% and temperature at 20-24°C. Mice were exposed to a 12-hour light-dark cycle. This has been described in in the 'Methods' section of the manuscript under 'Ethics statement and animals'.

Wild animals	No wild animals were used in this study.
Reporting on sex	The sex of mice used in each experiment is reported in the figure legend of all the main figures.
Field-collected samples	No field collected samples were used in this study.
Ethics oversight	All animal protocols were reviewed and approved by the Griffith University Animal Ethics Committee (GU-AEC) in accordance with the National Health and Medical Research Council (NHMRC) of Australia guidelines. Experimental protocols involving gene knock-out mice were reviewed and approved by Office of the Gene Technology Regulator (OGTR).

Note that full information on the approval of the study protocol must also be provided in the manuscript.

Clinical data

Policy information about clinical studies

All manuscripts should comply with the ICMJE guidelines for publication of clinical research and a completed CONSORT checklist must be included with all submissions.

Clinical trial registration	N/A
Study protocol	N/A
Data collection	N/A
Outcomes	N/A

Dual use research of concern

Policy information about dual use research of concern

Hazards

Could the accidental, deliberate or reckless misuse of agents or technologies generated in the work, or the application of information presented in the manuscript, pose a threat to:

No	Yes	
X	☐	Public health
X	☐	National security
X	☐	Crops and/or livestock
X	☐	Ecosystems
X	☐	Any other significant area

Experiments of concern

Does the work involve any of these experiments of concern:

No	Yes	
X	☐	Demonstrate how to render a vaccine ineffective
X	☐	Confer resistance to therapeutically useful antibiotics or antiviral agents
X	☐	Enhance the virulence of a pathogen or render a nonpathogen virulent
X	☐	Increase transmissibility of a pathogen
X	☐	Alter the host range of a pathogen
X	☐	Enable evasion of diagnostic/detection modalities
X	☐	Enable the weaponization of a biological agent or toxin
X	☐	Any other potentially harmful combination of experiments and agents

Plants

Seed stocks	N/A
Novel plant genotypes	N/A
Authentication	N/A

ChIP-seq

Data deposition

☐ Confirm that both raw and final processed data have been deposited in a public database such as GEO.

☐ Confirm that you have deposited or provided access to graph files (e.g. BED files) for the called peaks.

Data access links<br>*May remain private before publication.*	N/A
Files in database submission	N/A
Genome browser session<br>(e.g. UCSC)	N/A

Methodology

Replicates	N/A
Sequencing depth	N/A
Antibodies	N/A
Peak calling parameters	N/A
Data quality	N/A
Software	N/A

Flow Cytometry

Plots

Confirm that:

- [X] The axis labels state the marker and fluorochrome used (e.g. CD4-FITC).
- [X] The axis scales are clearly visible. Include numbers along axes only for bottom left plot of group (a 'group' is an analysis of identical markers).
- [X] All plots are contour plots with outliers or pseudocolor plots.
- [X] A numerical value for number of cells or percentage (with statistics) is provided.

Methodology

Sample preparation

Cell depletion studies: Macrophages were depleted using Clodronate Liposomes (ClLip; ClodLipBV, Haarlem, The Netherlands). ClLip was delivered intravenously (i.v.;100 µl at 0.5 mg/mouse) and i.n. (50 µl at 0.25 mg/mouse) for the depletion of systemic and alveolar macrophages. Control mice received PBS/liposomes. Mice were treated on days -3, -2, -1 and +1, relative to infection on day 0. Depletion was assessed by flow cytometry in lungs (alveolar macrophages; CD45+ F4/80+ CD11c+) and spleen (MHCII+ macrophages; CD45+ CD11b+ CD24- F4/80+ MHCII+).

Neutrophils were depleted using anti-Ly6G mAb (clone 1A8, BioXcell). Mice received 100 uL (0.5 mg/mouse) i.p. on days -3. -2 and -1, relative to infection on day 0. Depletion was confirmed by flow cytometry in lungs and spleen (CD45+ Ly6G+ CD11b+).

CD4+ T-cells were depleted using rat anti-mouse CD4 mAb (GK 1.5; Assay Matrix). Mice received 500 µl (0.215 mg/mouse) of ant-mouse CD4+ mAb i.p. or Rat IgG (isotype control) on days -4 and -1 before infection (day 0). CD4+ depletion was confirmed in cervical lymph nodes, NALT, lungs and spleen on days 0 and 3 post-mAb administration.

Assessment of TRM cells in the lungs: CD4+TRM cells were measured by identifying effector memory cells (CD62L- CD44+) followed by TRM cells (CD69+ CD103-).

A list of antibodies used in all flow cytometry experiments are available below -

CD45 30-F11 BUV395 BD Biosciences
CD3 145-2C11 PE-CF594 BD Biosciences
CD4 RM4-5 BV510 BD Biosciences
CD8a 53-6.7 PECy7 Invitrogen
F4/80 T45-2342 PE BD Biosciences
CD11b M1/70 BUV737 BD Biosciences
CD11c N418 AF488 BioLegend
CD24 M1/69 BV510 BD Biosciences
MHC11 (1A/1E) M5/114.15.2 AF700 BioLegend
Ly6G 1A8 BV510 BioLegend
CCR6 (CD196) 29-2L17 PE BioLegend
CD62L MEL-14 PerCP-Cy5.5 BD Biosciences
CD44 IM7 BV785 BioLegend
CD69 H1.2F3 BV421 BioLegend
CD103 M290 BV711 BD Biosciences
IL-17A TC11-18H10.1 APC BioLegend

Instrument

SR Fortessa Flow Cytometer (Becton Dickson)

Software

Data was analyzed on FlowJo v10 software for Macintosh and Windows

Cell population abundance

Macrophages – Supplementary Figure 9:

- Alveolar ($CD45^+CD11c^+F4/80^+$) = 7%
- Spleen ($CD45^+CD11b^+CD24^-MHCII^+F4/80^+$) = 26.9%

Neutrophils ($CD45^+CD11b^+Ly6G^+$) – Supplementary Figure 9:

- Lungs = 56.2%
- Spleen = 54.7%

CD4+ T-cells ($CD45^+CD3^+CD4^+$) – Supplementary Figure 6: abundance is ~30-75%

- Lungs = 56.7%
- Spleen = 69.3%
- NALT = 53.7%
- Lymph nodes = 68.3%

CD4$^+$ TRM cells (CD44$^+$CD62L$^-$CD103$^\pm$CD69$^+$) – Supplementary Figure 7:

- Lungs = 30%

Gating strategy

Gating strategy for the CD4$^+$ depletion experiment is presented Supplementary Figure 6.

Gating strategy for neutrophil and macrophage depletion experiments are presented in Supplementary Figure 9.

Gating strategy for the ICS (IL-17+ CD4+ cells in lungs) staining experiment is presented in Figure 2a.

Gating strategy for tissue resident memory (TRM) cells is presented in Supplementary Figure 7.

[X] Tick this box to confirm that a figure exemplifying the gating strategy is provided in the Supplementary Information.

Magnetic resonance imaging

Experimental design

Design type: N/A

Design specifications: N/A

Behavioral performance measures: N/A

Imaging type(s): N/A

Field strength: N/A

Sequence & imaging parameters: N/A

Area of acquisition: N/A

Diffusion MRI: [ ] Used [X] Not used

Preprocessing

Preprocessing software: N/A

Normalization: N/A

Normalization template: N/A

Noise and artifact removal: N/A

Volume censoring: N/A

Statistical modeling & inference

Model type and settings: N/A

Effect(s) tested: N/A

Specify type of analysis: [ ] Whole brain [ ] ROI-based [ ] Both

Statistic type for inference

(See Eklund et al. 2016)

N/A

Correction

N/A

Models & analysis

n/a	Involved in the study
X	☐ Functional and/or effective connectivity
X	☐ Graph analysis
X	☐ Multivariate modeling or predictive analysis

Functional and/or effective connectivity

N/A

Graph analysis

N/A

Multivariate modeling and predictive analysis

N/A

This checklist template is licensed under a Creative Commons Attribution 4.0 International License, which permits use, sharing, adaptation, distribution and reproduction in any medium or format, as long as you give appropriate credit to the original author(s) and the source, provide a link to the Creative Commons license, and indicate if changes were made. The images or other third party material in this article are included in the article's Creative Commons license, unless indicated otherwise in a credit line to the material. If material is not included in the article's Creative Commons license and your intended use is not permitted by statutory regulation or exceeds the permitted use, you will need to obtain permission directly from the copyright holder. To view a copy of this license, visit http://creativecommons.org/licenses/by/4.0/
